# Supplementary material for: Analytical Performance of the Commercial MucorGenius® Assay as Compared to an In-House qPCR Assay to Detect Mucorales DNA in Serum Specimens
Source: J Fungi (Basel). 2022 Jul 27;8(8):786. doi: 10.3390/jof8080786 (PMC9410016; doi:10.3390/jof8080786)
Supplement: Supplementary file 1 [file jof-08-00786-s001.zip › jof-1819174-supplementary.pdf]

Analytical performance of the commercial MucorGenius® assay as compared to an in-house qPCR assay to detect Mucorales DNA in serum specimens

Supplementary

Table S1. Patient characteristics and quantification cycle (Cq) of the two evaluated real-time PCR.

Patient	Underlaying disease	Classification[1]	IFD	Involved species	Sample	Positive qPCR	In-house qPCR[2]		MucorGenius®[2]
							initial	present	present
**Patient 1**	**SOT and DM**	**Probable**	**RM**	**na**	**Sample 01**	**Rmuc**	**39.24**	**50**	50
Patient 2	Critically ill burn	Proven	IWB	*Mucor circinelloides*	Sample 02	Muc	34.54	38.3	50
					Sample 03	Muc	35.08	39.86	50
					Sample 04	Muc	35.31	50	44.55
Patient 3	ALL	Proven	RM	na	Sample 05	Muc	35.81	37.79	50
					Sample 06	Muc	37.19	50	50
					Sample 07	Muc	37.94	50	50
					Sample 08	Muc	39.03	50	50
Patient 4	ALL	Probable	PM	na	Sample 09	Rmuc	33.24	35.22	39.2
Patient 5	Cirrhosis	Proven	GM	*Rhizopus oryzae*	Sample 10	Muc	33.56	37.76	50
Patient 6	Critically ill burn	Proven	IWB	na	Sample 11	Muc	39.92	50	50
Patient 7	AML	Probable	PM	na	Sample 12	Muc	34.66	39.56	36.72
					Sample 13	Rmuc	30.74	36.79	36.72
Patient 8	Critically ill burn	Probable	IWB	na	Sample 14	Muc	39.74	50	50
Patient 9	AML	Probable	PM	na	Sample 15	Acory	36.04	38.49	50
Patient 10	AML	Proven	RM	*Rhizopus arrhizus*	Sample 16	Muc	38.98	50	50
Patient 11	Critically ill burn	Proven	IWB	*Mucor circinelloides*	Sample 17	Muc	35.47	39.29	50
Patient 12	AML	Probable	RM	*Rhizopus arrhizus*	Sample 18	Muc	35.5	50	50
Patient 13	SOT	Probable	PM	na	Sample 19	Acory	37.86	38.57	50

					Sample 20	Acory	38.36	50	50
					Sample 21	Acory	39.05	50	50
					Sample 22	Acory	39.26	39.95	50
Patient 14	Critically ill burn	Proven	IWB	*Mucor circinelloides*	Sample 23	Muc	37.1	40.3	50
					Sample 24	Muc	38.21	50	50
Patient 15	Critically ill burn	Probable	IWB	na	Sample 25	Acory	36.81	50	50

[1] EORTC/MSG classification [11]; [2] Quantification cycle value.
Acory, *Lichtheimia* assay; ALL, acute lymphoid leukemia; AML, acute myeloid leukemia; DM, diabetes mellitus; GM, gastrointestinal mucormycosis; IWB, invasive wound mucormycosis; IFD, invasive fungal disease; Muc, *Mucor/Rhizopus* assay; na, not available; PM, pulmonary mucormycosis; qPCR, quantitative real-time polymerase chain reaction; RM, rhinocerebral mucormycosis; Rmuc, *Rhizomucor* assay; SOT, solid organ transplant.

Table S2. List of species used in the present study and their origin.

**Species**	**Strain origin[1]**
Actinomucor elegans	CNRMA 12.953
Apophysomyces elegans	CNRMA 18.469
Cunninghamella bertholletiae	CNRMA 18.729
Lichtheimia corymbifera	CNRMA 19.036
Mucor indicus	CNRMA 14.375
Rhizopus arrhizus var. *arrhizus*	CNRMA 19.615
Rhizopus arrhizus var. *delemar*	CNRMA 19.267
Rhizopus microsporus	CNRMA 14.351
Rhizomucor pusillus	CNRMA 14.832
Saksenaea vasiformis	CNRMA 8.1143
Syncephalastrum racemosum	CNRMA 17.439
Syncephalastrum monosporum	CBS 100.49
Aspergillus flavus	CNRMA 20.71
Aspergillus fumigatus	CBS 101.355
Penicillium digitatum	CNRMA 17.586

[1] CNRMA, French National Reference Center of invasive Mycoses and Antifungals; CBS, Fungal Biodiversity Centre.

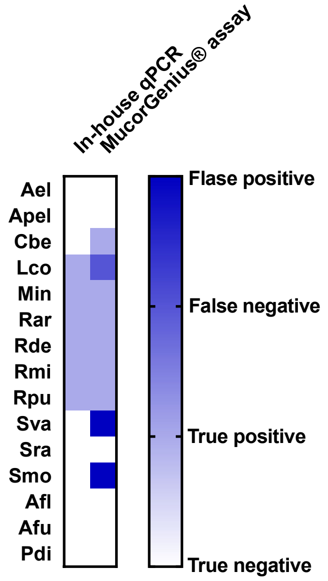

Figure S1. Assays performance based on spiked sera spiked with fungal DNA. *Actinomucor elegans* (Ael), *Apophysomyces elegans* (Apel), *Cunninghamella bertholletiae* (Cbe), *Lichtheimia corymbifera* (Lco), *Mucor indicus* (Min), *Rhizopus arrhizus* var. *arrhizus* (Rar), *Rhizopus arrhizus* var. *delemar* (Rde), *Rhizopus microsporus* (Rmi), *Rhizomucor pusillus* (Rpu), *Saksenaea vasiformis* (Sva), *Syncephalastrum racemosum* (Sra), *Syncephalastrum monosporum* (Smo) and *Aspergillus flavus* (Afl), *Aspergillus fumigatus* (Afu), *Penicillium digitatum* (Pdi).
